# 

**Published:** 1883-09

**Authors:** 


### CONTENTS.

ORIGINAL:

Precautionary Measures and Contra-Indications to the Use of Pressure by the Tampon in Diseases of the Pelvic Organs. By V. H. Taliaferro, M.D. .......... 705

Taliaferro's Hard Rubber Intra-Uterine Stem Pessary, Retro-Displacement Pessary, Universal Pessary and Tracheloraphy. By Geo. H. Noble, M.D .......... 712

The Outlook of Medicine and Surgery. No. 4. By J. McF. Gaston, M.D .......... 718

EXTRACTS:

Overwork The Treatment of Spasmodic Dysmenorrhœa – Recent Changes in the Method of Medical Instruction—Good Remedies Out of Fashion—Woman and her Ailments—An Obstetrical Expedient—Reflex Irritation—Tricycles for Practitioners—Death from the Sting of a Bee—Ergot as a Preventive of the Aurul Troubles Occasioned by Quinine and Salicylate of Soda—Detection of Stone in the Bladder of Children—Tuberculosis in Infants—Doctors—Poisoning from Eating Plum Stones—Castor Oil and Glycerine—Transfusion of Pure Water...725-750

EDITORIAL:

Practical Chemistry for Students and Physicians—Chapter Third, Continued from August No .......... 751

Exercises on Chapter Third .......... 755

Atlanta's Potable Water, Continued from August No .......... 756

Yankee Brass .......... 761

Pay Up .......... 761

BOOKS .......... 762

PAMPHLETS .......... 764

Index to Volume II .......... 765

